# Peptide Antiviral Strategies as an Alternative to Treat Lower Respiratory Viral Infections

**DOI:** 10.3389/fimmu.2019.01366

**Published:** 2019-06-21

**Authors:** Origène Nyanguile

**Affiliations:** HES-SO Valais-Wallis, Institute of Life Technologies, Sion, Switzerland

**Keywords:** escape mutation, peptides, fusion, antivirals, RSV, influenza

## Abstract

Lower respiratory infection caused by human pathogens such as influenza and respiratory syncytial virus (RSV) is a significant healthcare burden that must be addressed. The preferred options to achieve this goal are usually to develop vaccines for prophylaxis and to develop antiviral small molecules to treat infected patients with convenient, orally administrable drugs. However, developing a vaccine against RSV poses special challenges with the diminished immune system of infants and the elderly, and finding a universal flu vaccine is difficult because the product must target a large array of viral strains. On the other hand, the use of small-molecule antivirals can result in the emergence of resistant viruses as it has well-been reported for HIV, influenza, and hepatitis C virus (HCV). This paper reviews peptide antiviral strategies as an alternative to address these challenges. The discovery of influenza and RSV peptidic fusion inhibitors will be discussed and compared to small molecules in view of escape mutations. The importance of constraining peptides into macrocycles to improve both their inhibitory activity and pharmacological properties will be highlighted.

Lower respiratory infection is one of top four causes of human death worldwide (1), causing 3 million deaths in 2016. Among the pathogens responsible for these infections, influenza virus, respiratory syncytial virus (RSV), and pneumococcus are the most important causes. The year 2018 marks the 100th anniversary of one of the largest public health crises in modern history, the 1918 influenza pandemic known colloquially as “Spanish flu,” which at the time killed at least 2% of the Earth's population. Currently, influenza continues to represent a global threat because of the constant evolution of the virus into various strains and the increase of urbanization, mass migration, and global transports. The World Health Organization estimates that seasonal influenza is responsible for the death of about 500,000 persons every year (2); in contrast to the 1918 epidemic, which affected mainly the 20- to 40-year age group, influenza-associated hospitalizations are now the highest in children younger than 5 years and in the elderly (3). A meta-analysis estimated that, in 2008, there were 90 million new cases of influenza in children <5 years, of which 20 million developed acute lower respiratory infections (ALRIs) and 1 million developed severe ALRI (4). In 2010, there were 11.9 million hospital admissions due to severe ALRI and 3 million due to very severe ALRI (5). Currently, the mortality associated with influenza is higher in the elderly. A study covering the period from 1972 to 2003 in the United States estimates a yearly average of influenza-associated deaths of 2,680 individuals for those aged <65 years and 22,790 for those aged >65 years (6).

RSV caused almost 34 million cases of lower respiratory infections in children under 5 years of age in 2005 worldwide, resulting in approximately 100,000 deaths (7). Although RSV is not a large cause of mortality in developed countries, the virus creates a significant burden on the healthcare system, because of the high number of children under 5 years of age that must be hospitalized, accounting for 45% of the total children admissions. In the United States, it has been estimated that RSV is responsible for 86,000 children hospitalizations per year with an estimated cost of US$394 million (8). The virus is also an important source of infection in the elderly population and in bone marrow transplant recipients (9). It is estimated that RSV causes, on average, 17,358 deaths annually in the United States, 78% of which occur in adults aged 65 or older, resulting in 5% of the total elderly hospital admissions (10). However, contrary to influenza, RSV kills more in the pediatric population; RSV accounts for 6.7% of all deaths in infants aged 28–364 days (1).

To prevent another important influenza pandemic, an efficient vaccine is warranted. The current flu vaccines contain representative hemagglutinins (HAs) of H3N2, H1N1, and type B viruses. The vaccines are effective but the immunity takes time to develop; thus, they are of no use for the infected patient. Additionally, the vaccines must be reformulated each year because of the antigenic drift, and they are not effective when the formulation does not match the epidemic virus. For these reasons, a universal vaccine is sought to replace the seasonal vaccine by providing long-lasting protection against both seasonal and pandemic strains, thanks to the discovery of human broadly neutralizing antibodies (11, 12). In the case of RSV, despite decades of research, there are currently no licensed vaccines available. Current efforts are driven (i) toward immunizing pregnant women with a vaccine targeting the fusion F viral protein to protect neonates and young infants through trans-placental antibody transfer, and (ii) toward immunizing infants and young children with live attenuated vaccines (13). The discovery of the means to stabilize the metastable prefusion form of F (preF) (14) has been a major breakthrough in the field due to the following reasons. Firstly, preF neutralizing antibodies are more potent than postF neutralizing antibodies; secondly, preF contains six antigenic binding sites that can be used to generate antibodies (15), and lastly, the x-ray coordinates of preF can be utilized to perform an *in silico* study to engineer and screen for the best preF antigens in animals, prior to their application to human (14). Currently, 18 RSV vaccine trials and 21 preclinical development programs are under development (16). The most promising candidate is an RSV F nanoparticle-based vaccine of Novavax. This vaccine is under development against young infants, pregnant women, and the elderly. The maternal immunization phase 3 clinical trial is the most advanced (17, 18). The vaccine is a prefusogenic F protein encapsidated into a nanoparticle and complemented with an aluminum adjuvant to boost immunization. The primary endpoints of the phase 3 clinical trial have been met and the study will be unblinded shortly; the data are promising and suggest that the first RSV vaccine might be approved by the U.S. Food and Drug Administration soon. It will be valuable to see, in case of success, if the adjuvant is well tolerated by the fetus (and, by extension, by the young infants), and if the immunization of this vaccine can extend beyond 1–2 months. Persistence of maternal antibodies in the neonate may be too short to achieve reliable protection unless a very high titer of neutralizing antibodies is reached. Additionally, the timing of immunization can have an impact on level of transplacental antibody transfer from the mother to the fetus.

Since no vaccines are presently available to eradicate the seasonal flu, antiviral molecules are needed to treat the infected patients. The current standard of care against flu targets two proteins, the matrix-2 (M2), a proton-selective ion channel protein, or the neuraminidase (NA) protein. M2 enables the migration of H^+^ ions into the interior of virus particles, a process that takes place upon endosome acidification and is needed for virus uncoating to occur. NA cleaves the sialic acid that is used by the virus to bind to the host receptor, thereby allowing the release of the virus from the infected cell and further spreading in the host (19). The licensed drugs targeting M2 are amantadine (Symmetrel) and rimantadine (Flumadine), belonging to the class of adamantane derivatives, and the ones targeting NA are oseltamivir (Tamiflu), zanamivir (Relenza), and peramivir (Rapivab). In principle, these antivirals are universal and can be used against all strains of influenza virus. However, resistance strains have emerged in the last two decades and have become a serious issue. The use of the adamantane derivatives resulted in the appearance of several escape mutants in viruses isolated from man and avian in the transmembrane region of the M2 protein (20, 21). In particular, the S31N was shown to be present in all H3N2 and 15.5% of the H1N1 influenza A viruses worldwide by 2006 (22, 23). Resistance increased dramatically in the United States in a period of 10 years, starting from only 2% prevalence in 1999, to 15% in 2005, and finally 96.4% in 2006. In some Asian countries such as China, adamantane resistance was already detected in 70% of all virus isolates in 2004. On the other hand, the H274Y NA mutant resistant to oseltamivir and peramivir has naturally appeared in 2007 and is now present in virtually all H1N1 virus isolates (24). This still leaves the option of using the adamantanes to treat the infections due to H1N1 and oseltamivir to treat the infections due to H3N2. Even in the case that a virus resistant to both adamantanes and oseltamivir would appear to become predominant (25), zanamivir could still be used. However, because zanamivir is an inhalable drug, which requires the use of an unfriendly device to administer the compound, this option cannot be used to treat the pediatric population, the elderly, and patients with chronic airway disease such as asthma or chronic obstructive pulmonary disease (COPD) (26). In addition to this, a diagnostic tool must be available to identify quickly the subtype of the influenza virus for a prompt clinical decision.

Recently, a peptide-based strategy has been used to design peptidic macrocyclic compounds capable of inhibiting the fusion of influenza A group 1 viruses (27). Like broad neutralizing antibodies (bnAbs), these peptides aim at binding to the conserved HA stem, an approach that may reduce the likelihood of generating escape mutants. HA is a trimeric metastable protein, in which each subunit contains an HA1 and an HA2 subdomain linked by a disulfide bond. HA1 is a globular domain mediating binding of the virus to sialylated receptors at the surface of the host cell, and HA2 is an α-helical stem region mediating fusion of the viral membrane with the host cell membrane. Following binding to cell-surface receptors, the virus is internalized by endocytosis. The low pH of the endosomes (pH 5–6) triggers a major structural rearrangement of HA2. Each HA2 subunit monomer contains a long helix connected through an extended loop to a shorter helix. The C-terminus of the long helix is anchored to the viral membrane, and the fusion peptide is at the N-terminus of the short helix. This structure forms a six-helix bundle in trimeric HA2. As the shorter helix is antiparallel to the long helix, the fusion peptide is located next to the viral membrane in the prefusion form. Upon pH change, the extended loop rotates dramatically and merges with the long helix to position the fusion peptide at 180° from the viral membrane into the host cell endosomal membrane. This structural rearrangement is the driving force of the fusion between the viral and host cell membrane (28–30). A peptide binding to the prefusion HA stem region should prevent fusion of the virus with the host cell, thereby neutralizing the virus, as it is the case for bnAbs. To design such peptide, the authors combined discontinuous segments located at the complementary-determining regions (CDRs) and framework regions (FRs) of the bnAbs. Similar strategies had been used by others previously (31–35). CDRs are hypervariable loops of the antibody mediating binding to the antigen, and FRs are variable domains, assisting the CDRs to bind to the antigen. The x-ray structures of several bnAbs bound to the HA stem region were used to design the peptides described in this work. First, the x-ray structures of the bnAbs CR9114 and FI6v3 were compared, revealing a striking similarity between the binding mode of both bnAbs CDRs to HA stem. CR9114 is an antibody identified through screening combinatorial libraries constructed from human B cells, which neutralizes both influenza A and B viruses (36). FI6v3 is an antibody isolated through screening human peripheral blood plasma, which neutralizes all 16 influenza A subtypes (37). CR9114 uses residues of FR3, CDR1, and CDR3 to make hydrophobic contact with the HA stem, whereas FI6v3 uses mainly CDR3 (Figure 1). Therefore, the CDR3 sequence of FI6v3, a 14-mer peptide, was used as a starting point for the medicinal chemistry work. Firstly, Leu100B of FI6v3 was replaced with Glu to improve water solubility. Secondly, in order to improve the weak micromolar inhibitory activity of the resulting linear peptides, the peptide was constrained into a macrocycle through a lactam cyclization between the side chain of an ornithine and the carboxylic C-terminus end of the peptide. Thirdly, the polar unfavorable N-terminal arginine was replaced by a hydrophobic amino acid leading to a significant increase of affinity, and the peptide was further optimized by sequential amino acid replacement with non-natural amino acid bioisosteres designed on the basis of the binding of various bnAbs to the HA stem. Finally, these sequential changes were combined into one peptide, peptide P7, which was shown to bind to several influenza A subtypes of group 1, including the 2009 H1N1 pandemic and avian H5N1 strains. P7 is an 11-mer peptide with a molecular weight of 1,639 Da, containing three non-natural residues and one N-methylated amino acid in its sequence. P7 binds to the HA stem with an affinity of approximately 20 nM and is capable of inhibiting viral fusion in cells with an EC_50_ of 70 nM. *In vitro* stability of the P7 peptide was assessed in human and mouse plasma; no degradation could be observed over the course of the study (4 h), implying that the insertion of a macrocyclic constrain and non-natural amino acids confer enhanced stability on the peptide. The peptide displayed no cytotoxicity in Calu-3 lung-derived cell lines, and the pharmacokinetic profile in BALB/c is encouraging for further drug development. P7 has a *t*_1/2_ of ~ 2.7 h, and its clearance in the plasma is ~24 h. The design of peptide P7 is a remarkable example of the potential of peptide therapeutics and highlights the importance of constraining a peptide into a macrocycle to decrease the entropic cost required to go from the multiple possible conformations of unbound peptide to the rigid conformation of the receptor/peptide bound complex. Despite its small size, P7 is capable of burying almost the same surface, which is used by the anti-stem bnAbs to inhibit viral fusion; this surface is approximately 600 Å, and is too large to be accommodated by a small molecule. Of course, it remains to be seen if peptide P7 can be converted into an orally available drug or if, as for the treatment with Relenza, another mode of administration will need to be considered.

**Figure 1:**
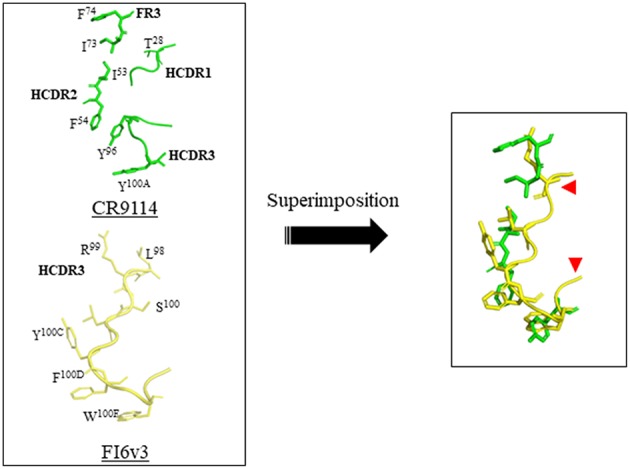
Design of a macrocyclic peptide binding to the HA stem binding site. **(Left)** The complementary-determining regions (CDRs) of the bnAbs CR9114 and FI6v3, which bind to HA stem, are shown. The HA protomers are not shown. The key determinants of CR9114 and FI6v3 are shown in green and yellow, respectively. The LCDR1 binding determinant of FI6v3 is not shown as it was not used in the design. **(Right)** overlay of FI6v3 HCDR3 with selected CR9114 key determinants, which was used as the basis for the design of peptide inhibitors. The red arrows depict approximately where the macrocyclic bride was inserted. This figure was prepared with Pymol using PDB ID 3ZTN and 4FQI for FI6v3 and CR9114, respectively.

Another example of pan inhibitor is P9, a peptide active against influenza A virus H1N1, H3N2, H5N1, H7N7, and H7N9 (38). P9 is a 30-amino-acid synthetic peptide derived from mouse β-defensin-4 (mBD4). The authors performed a series of N- and C-terminal truncation of mBD4, resulting in the identification of P9. The peptide contains two disulfide bonds and is rich in basic amino acids. P9 is active against various flu strains with IC_50_ values ranging from 450 nM to 1.43 μM. Experimental data suggest that P9 binds to viral glycoproteins and inhibits RNA replication through preventing the pH drop required to trigger viral fusion in the endosome, thanks to the high content of basic amino acids. Administration of P9 to mice prior to viral challenge showed that one-fifth of the dose was still present 8 h post-administration, suggesting that the presence of disulfide bonds confer a certain degree of proteolytic stability to the peptide despite the presence of only wild-type amino acids.

For RSV prospective therapies, resistance might become a problem with the neutralizing antibodies currently under development. The current standard of care consists of prophylactic treatment of at-risk infants with palivizumab, a monoclonal antibody against RSV F protein administered monthly as an injectable during the infection peak season (39, 40). Its limited efficacy and high cost limit its use to pre-term infants with bronchopulmonary dysplasia and chronic respiratory disease and newborns with congenital heart disease (8). As a result, 60% of at-risk children remain untreated, and no efficient therapy is available to treat the adult population (41). Recombinant antibodies targeting the antigenic sites of preF with longer half-life than palivizumab are currently developed to decrease the number of injection of the current standard of care, including REGN2222, ALX-0171 (42), and MEDI8897 (43, 44). Regeneron has recently reported the phase 3 clinical trial results of Suptavumab (REGN2222), a 5- to 10-fold more potent neutralizing antibody than palivizumab (45, 46). This trial failed to meet the primary endpoint against RSV B clinical isolates because of the emergence of the L172Q and S173L resistant mutants. This was completely unexpected since these variants, which had never been observed prior to 2015, appeared spontaneously over the last three seasons in genotype B patients enrolled in the study as well as in the placebo group. These results highlight that targeting a single antigenic epitope with neutralizing antibodies is risky, even if this appeared not to be a serious issue with palivizumab. As a result, MedImmune is carefully assessing the variants present in the population tested against the clinical candidate MEDI8897.

In principle, resistance should not be an issue for the treatment of young infants with antiviral drugs given that RSV is not a chronic disease; the viral load should decrease rapidly upon administration of the antiviral, leaving no time for the virus to mutate. However, the identification of the F140L and T400I escape mutants in the clinical phase 2a human challenge study of presatovir suggests that resistant circulating RSV strains could emerge (47). Additionally, resistance will become a problem for hematopoietic cell transplant (HCT) patients, since these patients need to go on therapy for a period of 6 months. The most advanced molecules that have been reported recently in clinical settings are Presatovir (GS-5806) (48), Lumicitabine (ALS-008176) (49), Ziresovir (AK0529), JNJ-53718678 (50), EDP-938 (51), and RV-521 (52). Presatovir, JNJ-53718678, and RV-521 are small-molecule fusion inhibitors targeting preF; Lumicitabine is a pro-drug nucleoside polymerase inhibitor; and EDP-938 is a replication inhibitor whose mechanism has not been disclosed. Johnson & Johnson has recently announced that they abandoned development of Lumicitabine (53). Presatovir and JNJ-53718678 are currently on hold; formulation issues must be addressed for JNJ-53718678. More importantly, the phase 2 clinical trial of Presatovir in HCT patients failed to meet its primary endpoint. It is likely that Gilead has also abandoned the phase 2b trial of Presatovir. Despite the fact that no patient was enrolled later than 5 days after the onset of the disease, this trial appeared to fail because the compound was not administered early enough. These results raise the question whether it will be ever possible to administer an antiviral molecule early enough to treat an infected patient.

All the small-molecule fusion inhibitors (including GS-5806, RV-521, and JNJ-53718678) that have been discovered to date against RSV most likely interact and bind to the same binding pocket of preF. This pocket is a threefold symmetric cavity, which is formed at the junction of three protomers of preF (54). The binding of small molecules to this site tethers the fusion peptide and the heptad repeat 2 (HR2) of F, thereby stabilizing the metastable preF state and preventing the conformational rearrangement of the fusion peptide and HR2 required for the fusion between the virus and the host cell membranes. These inhibitors can be viewed as a hand fidget spinner toy, in which each ring of the toy consists of one aromatic moiety of the inhibitor. This moiety makes an aromatic π-π stacking with Asp489 of HR2 and Phe140 of the fusion peptide. In some instances, as with JNJ-53718678, the inhibitor has only two rings and therefore fills only two lobes of this cavity (50). In other instances, as it is most likely the case for GS-5806, the inhibitor has three rings and is able to fill all three lobes of the binding pocket (54). As a result, it is likely that the emergence of an escape mutant would be resistant to all class of small-molecule fusion inhibitors. Consistent with this, cell culture resistance selection experiments resulted in escape mutations either at positions that directly contact the inhibitor (e.g., Phe140 and Phe488I) or at amino acid positions that are required to be displaced to accommodate the antiviral molecule (e.g., D498Y and L141W) (50, 54).

Peptides derived from the HR2 domain of F can also be used as fusion inhibitors. As these peptides target the transition between preF and postF (55, 56), the small-molecule escape mutants should not affect their inhibitory activity. RSV HR2 is a 49-amino-acid sequence that has been extensively characterized (Figure 2). HR2 is a largely unstructured peptide in aqueous solution folding into an α-helix upon binding to a trimeric HR1 coiled coil (57). A scan of synthetic peptides of 35 amino acids in length across the HR2 wild-type sequence resulted in the identification of T108 and T118, two peptides that were shown to be capable of blocking virus-mediated syncytia formation (58). Some efforts were made to reduce the length of the T108 sequence, but this turns out to be unsuccessful (59) as reducing the length of the peptide beyond 30 amino acids abrogates its activity. To improve the pharmacological properties of the peptides, T118 was modified into a series of stapled peptides (60). The stapled peptide technology relies on the incorporation of unnatural olefinic amino acids (UAA) at positions that will not interfere with the binding of the peptide to its target, and the subsequent cross-linking of these non-natural amino acids by Grubbs mediated ruthenium metathesis (61, 62). This results in the side-chain to side-chain incorporation of an all-hydrocarbon macrocyclic bridge, which can significantly increase the affinity of the peptide to the target, the proteolytic stability, and the cellular permeability through an endocytosis uptake mechanism. Because the staple is used to stabilize α-helical structures, the UAAs used for stapling are incorporated at i and i + 3, i and i + 4, or i and i + 7 positions within the sequence of the peptide, spanning one turn or two turns of the helix, respectively. “i” refers to the position where the first UAA is incorporated during the solid phase peptide synthesis (which occurs from the C- to the N-terminus); the second UAA can then be incorporated at 3, 4, or 7 amino acids away from the “i” position at its N-terminus. Bird et al. (60) tested some i, i + 4, and i, i + 7 single-stapled peptides within the N- and C-termini of T118 and evaluated the affinity of these analogs toward a 5HB (5-helix bundle) recombinant protein, a protein mimicking RSV postF, but lacking one of the three HR2 domains (59). When the resulting SAH-RSVF (stabilized α-helices of RSV F) peptides were tested for their affinity toward 5HB, they found that the i, i + 7 single-stapled peptides demonstrated notable enhanced binding activity, unlike the i, i + 4 single-stapled peptides, which had a binding affinity similar to the unmodified peptide. The best i, i + 7 peptides were combined into double-stapled peptides and tested in proteolytic resistance assays as well as in a cellular viral infectivity assay. This work led to the identification of SAH-RSVF_BD_ (Figure 2), which was then shown by intranasal administration with nanoparticles to be able to prevent RSV viral infection of BALBc mice. In a similar work (63), a stapled peptide scan across the HR2 sequence was performed with the aim to identify a minimal domain capable of disrupting the formation of postF. HR2 was divided into three overlapping subdomains, which were scanned for various stapling combinations. The binding affinity of the resulting peptides was assessed against recombinant 5 HB. In contrary to the 35-mer stapled peptide scan by Bird and colleagues (60), stapling did not improve the binding affinity of the single-stapled peptides, even with the i, i + 7 staple. Furthermore, the single-stapled peptides were all inactive in the cellular viral inhibition assay. However, the addition of a second staple restored the inhibitory activity of the peptide. Further optimization resulted in the discovery of peptide 4ca, which inhibits RSV infection of Hep-2 cells with an EC_50_ value of 0.59 μM (Figure 2). Peptide 4ca is a 20-mer sequence with a molecular weight of 2,441 Da, containing one i, i + 4 and one i, i + 3 staple. Despite its significantly shorter size, peptide 4ca inhibits RSV infection with a similar EC_50_ value to SAH-RSVF_BD_ in Hep-2 cells. Furthermore, peptide 4ca is highly stable to proteolytic digestion and is capable of decreasing RSV infection in the upper and lower respiratory tract of BALB/c mice following intranasal delivery of the peptide with no signs of apparent cytotoxicity and immunogenicity. Here, again, this work highlights how the insertion of macrocycle(s) can dramatically improve the potency and pharmacological properties of a peptide.

**Figure 2:**
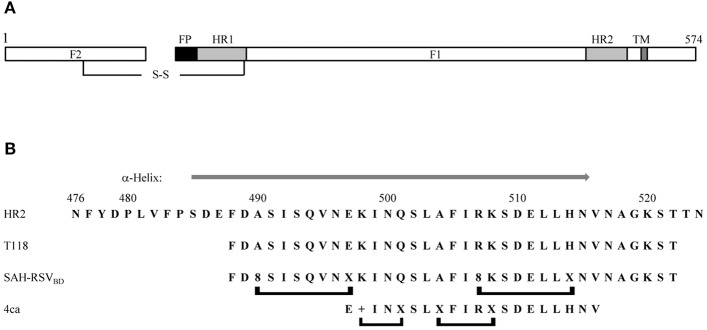
**(A)** Schematic representation of the fusion protein F. The F1 chain is linked to the F2 chain via a disulfide bridge. FP, fusion peptide; HR1, heptad repeat 1; HR2, heptad repeat 2; TM, transmembrane domain. **(B)** Comparison of the wild-type HR2 sequence with the inhibitory peptides T118, SAH-RSV_BD_, and peptide 4ca. A gray arrow above the HR2 sequence indicates the region folding as an α-helix in the x-ray structure of the postfusion structure (PDB accession number 1G2C). The brackets below the peptides indicate the position of the staples. +, R-pentenyl alanine; X, S-pentenyl alanine; 8, R-octenyl-alanine.

Peptides derived from the HR2 domain of F can also be used to develop pan inhibitors of other lower respiratory pathogens. A 36-mer peptide derived from the HR2 domain of human parainfluenza 3 (HIPV3) was found to inhibit HIPV3 as well as Hendra virus (Hev) and Nipah virus (Niv), two highly pathogenic viruses. Although the initial inhibitory activity of the 36-mer peptide was modest (IC_50_ = ~700 nM), a whole body of work was performed to significantly improve the potency of the peptide. Rather than performing the standard structural relationship activity studies (SAR), the authors added a cholesterol (Chol) moiety to improve the potency of the peptide. Such strategy was pioneered by Merck Research Laboratories during the development of enfurvitide follow-on products, a HIV gp41 fusion inhibitor (64). Through selectively enriching the peptide in the membrane, where viral fusion occurs, Chol tagging will improve the local concentration of the peptide, thereby increasing its antiviral potency. The Chol moiety is derivatized with a bromoacetyl function, which is used as an electrophile to react with a nucleophilic cysteine residue appended through a short GSG linker at the N- or C-terminus of the peptide, to form a thioether linkage (Figure 3). The addition of Chol to the 36-mer peptide (V-Chol) improved the potency 100-fold when it was attached at the C-terminus, but not at the N-terminus, consistent with the HIV work (68). Surprisingly, both V-Chol and the N-terminal Chol conjugates were even more potent against Hev and Niv (picomolar). Another interesting feature of this technology is that Chol appears to prolong the half-life of the peptide *in vivo*, through binding to serum proteins (69). More recently, the authors further explored the effect of the PEG spacer upon peptide inhibition (66). Briefly, the authors found that the increase in spacer length (i) is beneficial for the solubility and inhibitory activity of the Chol conjugate, (ii) enhances the kinetics of peptide insertion in lipid membranes, and (iii) enhances the susceptibility to protease degradation. Therefore, an optimum balance must be found to select the best PEG to insert in the Chol conjugate.

**Figure 3:**
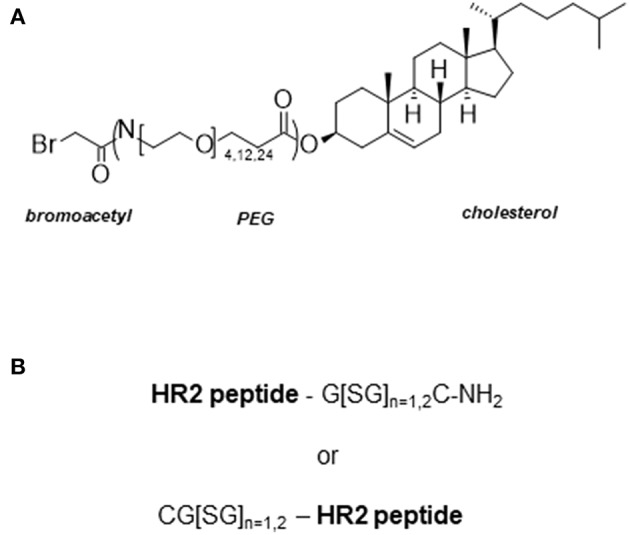
Cholesterol tagging of HR2 derived peptides. **(A)** Bromoacetyl cholesterol derivative used from the conjugation step to the HR2 peptide. The cholesterol with or without PEG is activated at its hydroxyl function with a bromoacetyl function as described in Santoprete et al. (65). The length of PEG spacer used in these studies was 4, 12, or 24. **(B)** The HR2 peptides are derived from HIV (64), HPIV3 (66), HeV and NiV (67). A cysteine residue appended at the N- or C-terminus of the HR2 peptide is used for chemoselective ligation of the peptide to the bromoacetyl cholesterol derivative. A Gly-Ser linker is inserted between the cysteine and the peptide to ensure sufficient spacing between the peptide and the lipid raft.

## Conclusion

The pharmaceutical industry has shown poor interest to develop peptide therapeutics due to their notoriously bad ADME (absorption, distribution, metabolism, elimination) properties. Peptides are rapidly degraded by proteases, can be immunogenic, do not efficiently cross the epithelial and skin barriers, and it is very challenging to confer oral bioavailability on these molecules (70). The bioavailability of peptides is typically <1%, whereas at least 20% is usually sought to make a drug. There are some exceptions such as cyclosporine A, an immunosuppressive undecapeptide with an impressive bioavailability of 26% (71), but in general, most marketed peptides such as Semaglutide, Liraglutide, and Exenatide are administered as injectables (72, 73). Additionally, the cost of goods to produce the active product ingredient (API) is higher for peptides than for small molecules. For all these reasons, the traditional medicinal chemist is usually unwilling to develop peptide therapeutics. However, given that many biological protein targets have binding sites that are too large to be accommodated by small molecules (74), and that it has become increasingly more difficult to develop orally available drugs, the field has seen a significant increase of injectable high-molecular-weight biological drugs. At the interface between these two classes of molecules, peptides are an alternative to target large protein–protein interaction sites. With the realization that macrocyclization can dramatically improve the binding affinity and proteolytic stability of peptides, there has been considerable effort devoted recently toward finding novel means to cyclize peptides (75). Furthermore, many labs are currently investigating the mechanisms conferring cellular permeability on cyclosporine A and other analogs with the aim of identifying novel tools to increase the bioavailability of peptide therapeutics (76–79).

Lower respiratory infections caused by influenza and RSV are a significant healthcare burden. Vaccines, neutralizing antibodies, bnAbs, and small molecules are currently under development to address this important unmet medical need. With the growing list of failed RSV drugs and vaccines, and the constant threat of a flu pandemic, peptides are an alternative to treat or prevent these diseases. Long-acting macrocyclic peptides may be used to target the influenza HA stem region or the mechanism of RSV F fusion, with the aim to develop a universal influenza treatment or a cheaper RSV prophylactic therapy with less susceptibility to escape mutations. It should not be forgotten that the HCV and HIV protease inhibitor drugs, simeprevir and darunavir, were peptides at the onset of the medicinal chemistry work.

## Author Contributions

The author confirms being the sole contributor of this work and has approved it for publication.

### Conflict of Interest Statement

The author declares that the research was conducted in the absence of any commercial or financial relationships that could be construed as a potential conflict of interest.

## References

[1] Lozano R Naghavi M Foreman K Lim S Shibuya K Aboyans V. Global and regional mortality from 235 causes of death for 20 age groups in 1990 and 2010: a systematic analysis for the Global Burden of Disease Study 2010. Lancet. (2012) 380:2095–128. 10.1016/S0140-6736(12)61728-0 23245604 PMC10790329

[2] WHO News/Fact sheets/Detail/Influenza (Seasonal). World Health Organization Available online at: https://www.who.int/en/news-room/fact-sheets/detail/influenza-(seasonal) (accessed April 10, 2019).

[3] Thompson WW Shay DK Weintraub E Brammer L Bridges CB Cox NJ. Influenza-associated hospitalizations in the United States. JAMA. (2004) 292:1333–40. 10.1001/jama.292.11.1333 15367555

[4] Nair H Brooks WA Katz M Roca A Berkley JA Madhi SA. Global burden of respiratory infections due to seasonal influenza in young children: a systematic review and meta-analysis. Lancet. (2011) 378:1917–30. 10.1016/S0140-6736(11)61051-9 22078723

[5] Nair H Simoes EA Rudan I Gessner BD Azziz-Baumgartner E Zhang JSF. Global and regional burden of hospital admissions for severe acute lower respiratory infections in young children in 2010: a systematic analysis. Lancet. (2013) 381:1380–90. 10.1016/S0140-6736(12)61901-1 23369797 PMC3986472

[6] Thompson WW Weintraub E Dhankhar P Cheng PY Brammer L Meltzer MI. Estimates of US influenza-associated deaths made using four different methods. Influenza Other Respir Viruses. (2009) 3:37–49. 10.1111/j.1750-2659.2009.00073.x 19453440 PMC4986622

[7] Nair H Nokes DJ Gessner BD Dherani M Madhi SA Singleton RJ. Global burden of acute lower respiratory infections due to respiratory syncytial virus in young children: a systematic review and meta-analysis. Lancet. (2010) 375:1545–55. 10.1016/S0140-6736(10)60206-1 20399493 PMC2864404

[8] Olszewska W Openshaw P. Emerging drugs for respiratory syncytial virus infection. Expert Opin Emerg Drugs. (2009) 14:207–17. 10.1517/14728210902946399 19453286 PMC2705842

[9] Collins PL Melero JA. Progress in understanding and controlling respiratory syncytial virus: still crazy after all these years. Virus Res. (2011) 162:80–99. 10.1016/j.virusres.2011.09.020 21963675 PMC3221877

[10] Thompson WW Shay DK Weintraub E Brammer L Cox N Anderson LJ. Mortality associated with influenza and respiratory syncytial virus in the United States. JAMA. (2003) 289:179–86. 12517228 10.1001/jama.289.2.179

[11] Cho A Wrammert J. Implications of broadly neutralizing antibodies in the development of a universal influenza vaccine. Curr Opin Virol. (2016) 17:110–5. 10.1016/j.coviro.2016.03.002 27031684 PMC4940123

[12] Impagliazzo A Milder F Kuipers H Wagner MV Zhu X Hoffman RM. A stable trimeric influenza hemagglutinin stem as a broadly protective immunogen. Science. (2015) 349:1301–6. 10.1126/science.aac7263 26303961

[13] Martin F Giersing B Hombach J Moorthy V Vekemans J WHO Preferred Characteristics for Respiratory Syncytial Virus (RSV) vaccines. World Health Organization (2017).

[14] McLellan JS Chen M Leung S Graepel KW Du X Yang Y. Structure of RSV fusion glycoprotein trimer bound to a prefusion-specific neutralizing antibody. Science. (2013) 340:1113–7. 10.1126/science.1234914 23618766 PMC4459498

[15] Graham BS. Vaccine development for respiratory syncytial virus. Curr Opin Virol. (2017) 23:107–12. 10.1016/j.coviro.2017.03.012 28525878 PMC5653266

[16] Mazur NI Higgins D Nunes MC Melero JA Langedijk AC Horsley N. The respiratory syncytial virus vaccine landscape: lessons from the graveyard and promising candidates. Lancet Infect Dis. (2018) 18:e295–311. 10.1016/S1473-3099(18)30292-5 29914800

[17] August A Glenn GM Kpamegan E Hickman SP Jani D Lu H. A Phase 2 randomized, observer-blind, placebo-controlled, dose-ranging trial of aluminum-adjuvanted respiratory syncytial virus F particle vaccine formulations in healthy women of childbearing age. Vaccine. (2017) 35:3749–59. 10.1016/j.vaccine.2017.05.045 28579233

[18] Novavax Progress Toward a Vaccine for Maternal Immunization to Prevent Respiratory Syncytial Virus Lower Respiratory Tract Illness (RSV LRTI) in Infants. Available online at: https://novavax.com/download/files/posters/11th-International-RSV-Symposium/Progress_Toward_a_Vaccine_for_Maternal_Immunization_to_Prevent_Respiratory_Syncytial_Virus_Lower_Respiratory_Tract_Illness_(RSV_LRTI)_in_Infants.pdf (accessed March 27, 2019).

[19] De Clercq E. Antiviral agents active against influenza A viruses. Nat Rev Drug Discov. (2006) 5:1015. 10.1038/nrd2175 17139286 PMC7097821

[20] Bright RA Medina MJ Xu X Perez-Oronoz G Wallis TR Davis XM. Incidence of adamantane resistance among influenza A (H3N2) viruses isolated worldwide from 1994 to 2005: a cause for concern. Lancet. (2005) 366:1175–81. 10.1016/S0140-6736(05)67338-2 16198766

[21] Bright RA Shay DK Shu B Cox NJ Klimov AI. Adamantane resistance among influenza A viruses isolated early during the 2005–2006 influenza season in the United States. JAMA. (2006) 295:891–4. 10.1001/jama.295.8.joc60020 16456087

[22] Deyde VM Xu X Bright RA Shaw M Smith CB Zhang Y. Surveillance of resistance to adamantanes among influenza A(H3N2) and A(H1N1) viruses isolated worldwide. J Infect Dis. (2007) 196:249–57. 10.1086/518936 17570112

[23] Duwe S. Influenza viruses—Antiviral therapy and resistance. GMS Infect Dis. (2017) 5:Doc04. 10.3205/id000030 30671326 PMC6301739

[24] Moscona A. Global transmission of oseltamivir-resistant influenza. N Engl J Med. (2009) 360:953–6. 10.1056/NEJMp0900648 19258250

[25] Sheu TG Fry AM Garten RJ Deyde VM Shwe T Bullion L. Dual resistance to adamantanes and oseltamivir among seasonal influenza A(H1N1) viruses: 2008-2010. J Infect Dis. (2011) 203:13–7. 10.1093/infdis/jiq005 21148491 PMC3086447

[26] Eiland LS Eiland EH. Zanamivir for the prevention of influenza in adults and children age 5 years and older. Ther Clin Risk Manag. (2007) 3:461–5. 18488077 PMC2386359

[27] Kadam RU Juraszek J Brandenburg B Buyck C Schepens WBG Kesteleyn B. Potent peptidic fusion inhibitors of influenza virus. Science. (2017) 358:496–502. 10.1126/science.aan0516 28971971 PMC5659926

[28] Bullough PA Hughson FM Skehel JJ Wiley DC. Structure of influenza haemagglutinin at the pH of membrane fusion. Nature. (1994) 371:37–43. 10.1038/371037a0 8072525

[29] Skehel JJ Wiley DC. Receptor binding and membrane fusion in virus entry: the influenza hemagglutinin. Annu Rev Biochem. (2000) 69:531–69. 10.1146/annurev.biochem.69.1.531 10966468

[30] Wiley DC Skehel JJ. The structure and function of the hemagglutinin membrane glycoprotein of influenza virus. Annu Rev Biochem. (1987) 56:365–94. 10.1146/annurev.bi.56.070187.002053 3304138

[31] Williams WV Kieber-Emmons T VonFeldt J Greene MI Weiner DB. Design of bioactive peptides based on antibody hypervariable region structures. Development of conformationally constrained and dimeric peptides with enhanced affinity. J Biol Chem. (1991) 266:5182–90. 2002053

[32] Levi M Sallberg M Ruden U Herlyn D Maruyama H Wigzell H. A complementarity-determining region synthetic peptide acts as a miniantibody and neutralizes human immunodeficiency virus type 1 *in vitro*. Proc Natl Acad Sci USA. (1993) 90:4374–8. 7685100 10.1073/pnas.90.10.4374 PMC46513

[33] Casset F Roux F Mouchet P Bes C Chardes T Granier C. A peptide mimetic of an anti-CD4 monoclonal antibody by rational design. Biochem Biophys Res Commun. (2003) 307:198–205. 10.1016/S0006-291X(03)01131-8 12850000

[34] Mutter M Hersperger R Gubernator K Muller K. The construction of new proteins: V. A template-assembled synthetic protein (TASP) containing both a 4-helix bundle and beta-barrel-like structure. Proteins. (1989) 5:13–21. 10.1002/prot.340050104 2748570

[35] Williams WV Guy HR Rubin DH Robey F Myers JN Kieber-Emmons T. Sequences of the cell-attachment sites of reovirus type 3 and its anti-idiotypic/antireceptor antibody: modeling of their three-dimensional structures. Proc Natl Acad Sci USA. (1988) 85:6488–92. 2457914 10.1073/pnas.85.17.6488 PMC281998

[36] Dreyfus C Laursen NS Kwaks T Zuijdgeest D Khayat R Ekiert DC. Highly conserved protective epitopes on influenza B viruses. Science. (2012) 337:1343–8. 10.1126/science.1222908 22878502 PMC3538841

[37] Corti D Voss J Gamblin SJ Codoni G Macagno A Jarrossay D. A neutralizing antibody selected from plasma cells that binds to group 1 and group 2 influenza A hemagglutinins. Science. (2011) 333:850–6. 10.1126/science.1205669 21798894

[38] Zhao H Zhou J Zhang K Chu H Liu D Poon VK. A novel peptide with potent and broad-spectrum antiviral activities against multiple respiratory viruses. Sci Rep. (2016) 6:22008. 10.1038/srep22008 26911565 PMC4766503

[39] Wu H Pfarr DS Losonsky GA Kiener PA. Immunoprophylaxis of RSV infection: advancing from RSV-IGIV to palivizumab and motavizumab. Curr Top Microbiol Immunol. (2008) 317:103–23. 17990791 10.1007/978-3-540-72146-8_4

[40] Wu H Pfarr DS Tang Y An LL Patel NK Watkins JD. Ultra-potent antibodies against respiratory syncytial virus: effects of binding kinetics and binding valence on viral neutralization. J Mol Biol. (2005) 350:126–44. 10.1016/j.jmb.2005.04.049 15907931

[41] Wang D Cummins C Bayliss S Sandercock J Burls A. Immunoprophylaxis against respiratory syncytial virus (RSV) with palivizumab in children: a systematic review and economic evaluation. Health Technol Assess. (2008) 12:1–86. 10.3310/hta12360 19049692

[42] Larios Mora A Detalle L Gallup JM Van Geelen A Stohr T Duprez L. Delivery of ALX-0171 by inhalation greatly reduces respiratory syncytial virus disease in newborn lambs. MAbs. (2018) 10:778–95. 10.1080/19420862.2018.1470727 29733750 PMC6150622

[43] Zhu Q McLellan JS Kallewaard NL Ulbrandt ND Palaszynski S Zhang J. A highly potent extended half-life antibody as a potential RSV vaccine surrogate for all infants. Sci Transl Med. (2017) 9:eaaj1928. 10.1126/scitranslmed.aaj1928 28469033

[44] Griffin MP Khan AA Esser MT Jensen K Takas T Kankam MK. Safety, tolerability, and pharmacokinetics of MEDI8897, the respiratory syncytial virus prefusion F-targeting monoclonal antibody with an extended half-life, in healthy adults. Antimicrob Agents Chemother. (2017) 61:e01714–16. 10.1128/AAC.01714-16 27956428 PMC5328523

[45] Gilman MS Castellanos CA Chen M Ngwuta JO Goodwin E Moin SM. Rapid profiling of RSV antibody repertoires from the memory B cells of naturally infected adult donors. Sci Immunol. (2016) 1:eaaj1879. 10.1126/sciimmunol.aaj1879 28111638 PMC5244814

[46] Clinicaltrials.gov. Study to Evaluate the Efficacy and Safety of Suptavumab (REGN2222) for the Prevention of Medically Attended RSV (Respiratory Syncytial Virus) Infection in Preterm Infants. Available online at: https://www.clinicaltrials.gov/ct2/show/NCT02325791?term=REGN2222&rank=2 (accessed April 11, 2019).

[47] Jordan R Stray K Anderson F Perron M Mackman R Svarovskaia E Analysis of Presatovir (GS-5806) Resistance Emergence in Human Healthy Adult Subjects Experimentally Infected with Respiratory Syncytial Virus. (2015). San Diego, CA: IDWeek. (accessed October 7, 2011).

[48] DeVincenzo JP Whitley RJ Mackman RL Scaglioni-Weinlich C Harrison L Farrell E. Oral GS-5806 activity in a respiratory syncytial virus challenge study. N Engl J Med. (2014) 371:711–22. 10.1056/NEJMoa1401184 25140957

[49] DeVincenzo JP McClure MW Symons JA Fathi H Westland C Chanda S. Activity of oral ALS-008176 in a respiratory syncytial virus challenge study. N Engl J Med. (2015) 373:2048–58. 10.1056/NEJMoa1413275 26580997

[50] Roymans D Alnajjar SS Battles MB Sitthicharoenchai P Furmanova-Hollenstein P Rigaux P. Therapeutic efficacy of a respiratory syncytial virus fusion inhibitor. Nat Commun. (2017) 8:167. 10.1038/s41467-017-00170-x 28761099 PMC5537225

[51] Rhodin M EDP-938, a novel non-fusion replication inhibitor of RSV, displays high barrier for resistance to RSV *in vitro*. In: 11th International Respiratory Syncytial Virus Symposium, Omni Grove Park Inn. Asheville, NC (2018).

[52] DeVincenzo JP Kim-Hoehamer Y-I Tait D Scott C Dowey R Mathews N Low rate of resistance to the RSV fusion inhibitor RV521 in human infection. In: 11th International Respiratory Syncytial Virus Symposium, Omni Grove Park Inn. Asheville, NC (2018).

[53] FierceBiotech J&J Dumps Alios RSV Drug, Wiping Remaining $900M off Value. Available online at: https://www.fiercebiotech.com/biotech/j-j-dumps-alios-rsv-drug-wiping-remaining-900m-off-value?mkt_tok=eyJpIjoiTlRVNE9XWTVNRFEyTlRZNSIsInQiOiI4c3hIMDBnWVhqekdzdUZVVk11Wm9vXC81ZXJ6SnpXMXhocmJFZmtUWklBK3YzekVVM1ZtYW9OY0ZldHF4QmpWcVF2YlZcL2V4TW9kY2dOaXlURWFxOExRU3JocXdqYURYT1dQeGg5RGNiN0Q5VGJKV1JrZytTVVBreUNpd3gxVmJrIn0%3D&mrkid=52731424&utm_medium=nl&utm_source=internal (accessed April 12, 2019).

[54] Battles MB Langedijk JP Furmanova-Hollenstein P Chaiwatpongsakorn S Costello HM Kwanten L. Molecular mechanism of respiratory syncytial virus fusion inhibitors. Nat Chem Biol. (2016) 12:87–93. 10.1038/nchembio.1982 26641933 PMC4731865

[55] Zhao X Singh M Malashkevich VN Kim PS. Structural characterization of the human respiratory syncytial virus fusion protein core. Proc Natl Acad Sci USA. (2000) 97:14172–7. 10.1073/pnas.260499197 11106388 PMC18890

[56] McLellan JS Yang Y Graham BS Kwong PD. Structure of respiratory syncytial virus fusion glycoprotein in the postfusion conformation reveals preservation of neutralizing epitopes. J Virol. (2011) 85:7788–96. 10.1128/JVI.00555-11 21613394 PMC3147929

[57] Matthews JM Young TF Tucker SP Mackay JP. The core of the respiratory syncytial virus fusion protein is a trimeric coiled coil. J Virol. (2000) 74:5911–20. 10.1128/jvi.74.13.5911-5920.2000 10846072 PMC112087

[58] Lambert DM Barney S Lambert AL Guthrie K Medinas R Davis DE. Peptides from conserved regions of paramyxovirus fusion (F) proteins are potent inhibitors of viral fusion. Proc Natl Acad Sci USA. (1996) 93:2186–91. 8700906 10.1073/pnas.93.5.2186 PMC39932

[59] Park M Matsuura H Lamb RA Barron AE Jardetzky TS. A fluorescence polarization assay using an engineered human respiratory syncytial virus F protein as a direct screening platform. Anal Biochem. (2011) 409:195–201. 10.1016/j.ab.2010.10.020 20971054 PMC3462168

[60] Bird GH Boyapalle S Wong T Opoku-Nsiah K Bedi R Crannell WC. Mucosal delivery of a double-stapled RSV peptide prevents nasopulmonary infection. J Clin Invest. (2014) 124:2113–24. 10.1172/JCI71856 24743147 PMC4001541

[61] Kim YW Grossmann TN Verdine GL. Synthesis of all-hydrocarbon stapled α-helical peptides by ring-closing olefin metathesis. Nat Protoc. (2011) 6:761–71. 10.1038/nprot.2011.324 21637196

[62] Verdine GL Hilinski GJ. Stapled peptides for intracellular drug targets. Methods Enzymol. (2012) 503:3–33. 10.1016/B978-0-12-396962-0.00001-X 22230563

[63] Gaillard V Galloux M Garcin D Eléouët JF Le Goffic R Larcher T. A short double-stapled peptide inhibits respiratory syncytial virus entry and spreading. Antimicrob Agents Chemother. (2017) 61:e02241–16. 10.1128/AAC.02241-16 28137809 PMC5365662

[64] Ingallinella P Bianchi E Ladwa NA Wang YJ Hrin R Veneziano M. Addition of a cholesterol group to an HIV-1 peptide fusion inhibitor dramatically increases its antiviral potency. Proc Natl Acad Sci USA. (2009) 106:5801–6. 10.1073/pnas.0901007106 19297617 PMC2667053

[65] Santoprete A Capito E Carrington PE Pocai A Finotto M Langella A. DPP-IV-resistant, long-acting oxymtomodulin derivatives. J Pept Sci. (2010) 17:270–80. 10.1002/psc.1328 21294225

[66] Mathieu C Augusto MT Niewiesk S Horvat B Palermo LM Sanna G. Broad spectrum antiviral activity for paramyxoviruses is modulated by biophysical properties of fusion inhibitory peptides. Sci Rep. (2017) 7:43610. 10.1038/srep43610 28344321 PMC5361215

[67] Porotto M Rockx B Yokoyama CC Talekar A Devito I Palermo LM. Inhibition of Nipah virus infection *in vivo*: targeting an early stage of paramyxovirus fusion activation during viral entry. PLoS Pathog. (2010) 6:e1001168. 10.1371/journal.ppat.1001168 21060819 PMC2965769

[68] Porotto M Yokoyama CC Palermo LM Mungall B Aljofan M Cortese R. Viral entry inhibitors targeted to the membrane site of action. J Virol. (2010) 84:6760–8. 10.1128/JVI.00135-10 20357085 PMC2903269

[69] Pessi A Langella A Capitò E Ghezzi S Vicenzi E Poli G. A general strategy to endow natural fusion-protein-derived peptides with potent antiviral activity. PLoS ONE. (2012) 7:e36833. 10.1371/journal.pone.0036833 22666328 PMC3353973

[70] Lau JL Dunn MK. Therapeutic peptides: historical perspectives, current development trends, and future directions. Bioorg Med Chem. (2018) 26:2700–7. 10.1016/j.bmc.2017.06.052 28720325

[71] Schreiber SL Crabtree GR. The mechanism of action of cyclosporin A and FK506. Immunol Today. (1992) 13:136–42. 10.1016/0167-5699(92)90111-J 1374612

[72] Kaspar AA Reichert JM. Future directions for peptide therapeutics development. Drug Discov Today. (2013) 18:807–17. 10.1016/j.drudis.2013.05.011 23726889

[73] Saladin PM Zhang BD Reichert JM. Current trends in the clinical development of peptide therapeutics. IDrugs. (2009) 12:779–84. 19943221

[74] Clackson T Wells JA. A hot spot of binding energy in a hormone–receptor interface. Science. (1995) 267:383–6. 7529940 10.1126/science.7529940

[75] Bock JE Gavenonis J Kritzer JA. Getting in shape: controlling peptide bioactivity and bioavailability using conformational constraints. ACS Chem Biol. (2013) 8:488–99. 10.1021/cb300515u 23170954 PMC4847942

[76] Goetz GH Farrell W Shalaeva M Sciabola S Anderson D Yan J. High throughput method for the indirect detection of intramolecular hydrogen bonding. J Med Chem. (2014) 57:2920–9. 10.1021/jm401859b 24641175

[77] Rezai T Bock JE Zhou MV Kalyanaraman C Lokey RS Jacobson MP. Conformational flexibility, internal hydrogen bonding, and passive membrane permeability: successful in silico prediction of the relative permeabilities of cyclic peptides. J Am Chem Soc. (2006) 128:14073–80. 10.1021/ja063076p 17061890

[78] Rezai T Yu B Millhauser GL Jacobson MP Lokey RS. Testing the conformational hypothesis of passive membrane permeability using synthetic cyclic peptide diastereomers. J Am Chem Soc. (2006) 128:2510–1. 10.1021/ja0563455 16492015

[79] White TR Renzelman CM Rand AC Rezai T McEwen CM Gelev VM. On-resin N-methylation of cyclic peptides for discovery of orally bioavailable scaffolds. Nat Chem Biol. (2011) 7:810–7. 10.1038/nchembio.664 21946276 PMC3210067

